# A new *Z*-eigenvalue localization set for tensors

**DOI:** 10.1186/s13660-017-1363-6

**Published:** 2017-04-21

**Authors:** Jianxing Zhao

**Affiliations:** College of Data Science and Information Engineering, Guizhou Minzu University, Guiyang, Guizhou 550025 P.R. China

**Keywords:** 15A18, 15A69, *Z*-eigenvalue, localization set, nonnegative tensors, spectral radius, weakly symmetric

## Abstract

A new *Z*-eigenvalue localization set for tensors is given and proved to be tighter than those in the work of Wang *et al.* (Discrete Contin. Dyn. Syst., Ser. B 22(1):187-198, 2017). Based on this set, a sharper upper bound for the *Z*-spectral radius of weakly symmetric nonnegative tensors is obtained. Finally, numerical examples are given to verify the theoretical results.

## Introduction

For a positive integer *n*, $n\geq2$, *N* denotes the set $\{1,2,\ldots ,n\}$. $\mathbb{C}$ ($\mathbb{R}$) denotes the set of all complex (real) numbers. We call $\mathcal{A}=(a_{i_{1}i_{2}\cdots i_{m}})$ a real tensor of order *m* dimension *n*, denoted by $\mathbb{R}^{[m,n]}$, if

$$a_{i_{1}i_{2}\cdots i_{m}}\in{\mathbb{R}}, $$

where $i_{j}\in{N}$ for $j=1,2,\ldots,m$. $\mathcal{A}$ is called nonnegative if $a_{i_{1}i_{2}\cdots i_{m}}\geq0$. $\mathcal{A}=(a_{i_{1}\cdots i_{m}})\in\mathbb{R}^{[m,n]}$ is called symmetric [2] if

$$ a_{i_{1}\cdots i_{m}}=a_{\pi(i_{1}\cdots i_{m})},\quad \forall\pi\in\Pi _{m}, $$

where $\Pi_{m}$ is the permutation group of *m* indices. $\mathcal{A}=(a_{i_{1}i_{2}\cdots i_{m}})\in\mathbb{R}^{[m,n]}$ is called weakly symmetric [3] if the associated homogeneous polynomial

$$\mathcal{A}x^{m}=\sum_{i_{1},i_{2},\ldots,i_{m}\in N}a_{i_{1}i_{2}\cdots i_{m}}x_{i_{1}}x_{i_{2}} \cdots x_{i_{m}} $$

satisfies $\nabla\mathcal{A}x^{m}=m\mathcal{A}x^{m-1}$. It is shown in [3] that a symmetric tensor is necessarily weakly symmetric, but the converse is not true in general.

Given a tensor $\mathcal{A}=(a_{i_{1}\cdots i_{m}})\in\mathbb {R}^{[m,n]}$, if there are $\lambda\in\mathbb{C}$ and $x=(x_{1},x_{2}\cdots,x_{n})^{T}\in\mathbb{C}^{n}\backslash\{0\}$ such that

$$\mathcal{A}x^{m-1}=\lambda x \quad \text{and}\quad x^{T}x=1, $$

then *λ* is called an *E*-eigenvalue of $\mathcal{A}$ and *x* an *E*-eigenvector of $\mathcal{A}$ associated with *λ*, where $\mathcal{A}x^{m-1}$ is an *n* dimension vector whose *i*th component is

$$\bigl(\mathcal {A}x^{m-1}\bigr)_{i}=\sum _{i_{2},\ldots,i_{m}\in N} a_{ii_{2}\cdots i_{m}}x_{i_{2}}\cdots x_{i_{m}}. $$

If *λ* and *x* are all real, then *λ* is called a *Z*-eigenvalue of $\mathcal {A}$ and *x* a *Z*-eigenvector of $\mathcal{A}$ associated with *λ*; for details, see [2, 4].

Let $\mathcal{A}=(a_{i_{1}\cdots i_{m}})\in{\mathbb{R}}^{[m,n]}$. We define the *Z*-spectrum of $\mathcal{A}$, denoted $\sigma(\mathcal{A})$ to be the set of all *Z*-eigenvalues of $\mathcal{A}$. Assume $\sigma(\mathcal{A})\neq0$, then the *Z*-spectral radius [3] of $\mathcal{A}$, denoted $\varrho (\mathcal{A})$, is defined as

$$\varrho(\mathcal{A}):=\sup\bigl\{ |\lambda|:\lambda\in\sigma(\mathcal{A})\bigr\} . $$

Recently, much literature has focused on locating all *Z*-eigenvalues of tensors and bounding the *Z*-spectral radius of nonnegative tensors in [1, 5–10]. It is well known that one can use eigenvalue inclusion sets to obtain the lower and upper bounds of the spectral radius of nonnegative tensors; for details, see [1, 11–14]. Therefore, the main aim of this paper is to give a tighter *Z*-eigenvalue inclusion set for tensors, and use it to obtain a sharper upper bound for the *Z*-spectral radius of weakly symmetric nonnegative tensors.

In 2017, Wang *et al.* [1] established the following Gers̆gorin-type *Z*-eigenvalue inclusion theorem for tensors.

### Theorem 1

[1], Theorem 3.1

*Let* $\mathcal{A}=(a_{i_{1}\cdots i_{m}})\in{\mathbb{R}}^{[m,n]}$. *Then*

$$ \sigma(\mathcal{A})\subseteq\mathcal{K}(\mathcal{A})=\bigcup _{i\in {N}}\mathcal{K}_{i}(\mathcal{A}), $$

*where*

$$ \mathcal{K}_{i}(\mathcal{A})=\bigl\{ z\in{\mathbb{C}}:|z|\leq R_{i}(\mathcal {A})\bigr\} ,\qquad R_{i}(\mathcal{A})=\sum _{i_{2},\ldots, i_{m}\in N}|a_{ii_{2}\cdots i_{m}}|. $$

To get a tighter *Z*-eigenvalue inclusion set than $\mathcal{K}(\mathcal{A})$, Wang *et al.* [1] gave the following Brauer-type *Z*-eigenvalue localization set for tensors.

### Theorem 2

[1], Theorem 3.2

*Let* $\mathcal{A}=(a_{i_{1}\cdots i_{m}})\in{\mathbb{R}}^{[m,n]}$. *Then*

$$ \sigma(\mathcal{A})\subseteq\mathcal{L}(\mathcal{A}) =\bigcup _{i\in N}\bigcap_{j\in N,j\neq i} \mathcal{L}_{i,j}(\mathcal{A}), $$

*where*

$$\mathcal{L}_{i,j}(\mathcal{A})= \bigl\{ z\in{\mathbb{C}}: \bigl(|z|- \bigl(R_{i}(\mathcal{A})-|a_{ij\cdots j}|\bigr) \bigr)|z| \leq|a_{ij\cdots j}|R_{j}(\mathcal{A}) \bigr\} . $$

In this paper, we continue this research on the *Z*-eigenvalue localization problem for tensors and its applications. We give a new *Z*-eigenvalue inclusion set for tensors and prove that the new set is tighter than those in Theorem 1 and Theorem 2. As an application of this set, we obtain a new upper bound for the *Z*-spectral radius of weakly symmetric nonnegative tensors, which is sharper than some existing upper bounds.

## Main results

In this section, we give a new *Z*-eigenvalue localization set for tensors, and establish the comparison between this set with those in Theorem 1 and Theorem 2. For simplification, we denote

$$\begin{aligned}& \Delta_{j}=\bigl\{ (i_{2},i_{3},\ldots, i_{m}): i_{k}=j\mbox{ for some }k\in\{2,\ldots,m\}, \mbox{where }j,i_{2},\ldots, i_{m}\in N\bigr\} , \\& \overline{\Delta}_{j}=\bigl\{ (i_{2},i_{3}, \ldots, i_{m}): i_{k}\neq j\mbox{ for any }k\in\{ 2,\ldots,m \}, \mbox{where }j,i_{2},\ldots, i_{m}\in N\bigr\} . \end{aligned}$$

For $\forall i,j\in N, j\neq i$, let

$$ r_{i}^{\Delta_{j}}(\mathcal{A})=\sum_{(i_{2},\ldots,i_{m})\in\Delta _{j}}|a_{ii_{2}\cdots i_{m}}|, \qquad r_{i}^{\overline{\Delta}_{j}}(\mathcal{A})=\sum _{(i_{2},\ldots,i_{m})\in \overline{\Delta}_{j}}|a_{ii_{2}\cdots i_{m}}|. $$

Then $R_{i}(\mathcal{A})=r_{i}^{\Delta_{j}}(\mathcal{A})+r_{i}^{\overline{\Delta }_{j}}(\mathcal{A})$.

### Theorem 3

*Let* $\mathcal{A}=(a_{i_{1}\cdots i_{m}})\in{\mathbb{R}}^{[m,n]}$. *Then*

$$ \sigma(\mathcal{A})\subseteq\Psi(\mathcal{A})=\bigcup _{i\in N}\bigcap_{j\in N, j\neq i} \Psi_{i,j}(\mathcal{A}), $$

*where*

$$ \Psi_{i,j}(\mathcal{A})= \bigl\{ z\in\mathbb{C}: \bigl(|z|-r_{i}^{\overline {\Delta}_{j}}( \mathcal{A}) \bigr)|z|\leq r_{i}^{\Delta_{j}}(\mathcal {A})R_{j}(\mathcal{A}) \bigr\} . $$

### Proof

Let *λ* be a *Z*-eigenvalue of $\mathcal{A}$ with corresponding *Z*-eigenvector $x=(x_{1},\ldots,x_{n})^{T}\in{\mathbb{C}}^{n}\backslash \{0\}$, *i.e.*,

1 $$ \mathcal{A}x^{m-1}=\lambda x,\quad \text{and}\quad \|x \|_{2}=1. $$

Assume $|x_{t}|=\max_{i \in N}|x_{i}|$, then $0<|x_{t}|^{m-1}\leq|x_{t}|\leq1$. For $\forall j\in N$, $j\neq t$, from (1), we have

$$ \lambda x_{t}=\sum_{(i_{2},\ldots, i_{m})\in\Delta_{j}}a_{ti_{2}\cdots i_{m}}x_{i_{2}} \cdots x_{i_{m}} +\sum_{(i_{2},\ldots, i_{m})\in\overline{\Delta}_{j}}a_{ti_{2}\cdots i_{m}}x_{i_{2}} \cdots x_{i_{m}}. $$

Taking the modulus in the above equation and using the triangle inequality give

$$\begin{aligned} |\lambda||x_{t}| \leq& \sum_{(i_{2},\ldots, i_{m})\in\Delta _{j}}|a_{ti_{2}\cdots i_{m}}||x_{i_{2}}| \cdots|x_{i_{m}}| +\sum_{(i_{2},\ldots, i_{m})\in\overline{\Delta}_{j}}|a_{ti_{2}\cdots i_{m}}||x_{i_{2}}| \cdots|x_{i_{m}}| \\ \leq& \sum_{(i_{2},\ldots, i_{m})\in\Delta_{j}}|a_{ti_{2}\cdots i_{m}}||x_{j}| +\sum_{(i_{2},\ldots, i_{m})\in\overline{\Delta}_{j}}|a_{ti_{2}\cdots i_{m}}||x_{t}| \\ =&r_{t}^{\Delta_{j}}(\mathcal{A})|x_{j}|+r_{t}^{\overline{\Delta}_{j}}( \mathcal{A})|x_{t}|, \end{aligned}$$

*i.e.*,

2 $$ \bigl(|\lambda|-r_{t}^{\overline{\Delta}_{j}}(\mathcal{A}) \bigr)|x_{t}|\leq r_{t}^{\Delta _{j}}(\mathcal{A})|x_{j}|. $$

If $|x_{j}|=0$, by $|x_{t}|>0$, we have $|\lambda|-r_{t}^{\overline{\Delta}_{j}}(\mathcal{A})\leq0$. Then

$$ \bigl(|\lambda|-r_{t}^{\overline{\Delta}_{j}}(\mathcal{A})\bigr)|\lambda|\leq0 \leq r_{t}^{\Delta_{j}}(\mathcal{A})R_{j}(\mathcal{A}). $$

Obviously, $\lambda\in\Psi_{t,j}(\mathcal{A})$. Otherwise, $|x_{j}|>0$. From (1), we have

3 $$ |\lambda||x_{j}|\leq\sum_{i_{2},\ldots, i_{m}\in N}|a_{ji_{2}\cdots i_{m}}||x_{i_{2}}| \cdots|x_{i_{m}}| \leq\sum_{i_{2},\ldots, i_{m}\in N}|a_{ji_{2}\cdots i_{m}}||x_{t}|^{m-1} \leq R_{j}(\mathcal{A})|x_{t}|. $$

Multiplying (2) with (3) and noting that $|x_{t}||x_{j}|>0$, we have

$$ \bigl(|\lambda|-r_{t}^{\overline{\Delta}_{j}}(\mathcal{A})\bigr)|\lambda|\leq r_{t}^{\Delta_{j}}(\mathcal{A})R_{j}(\mathcal{A}), $$

which implies that $\lambda\in\Psi_{t,j}(\mathcal{A})$. From the arbitrariness of *j*, we have $\lambda\in\bigcap_{j\in N, j\neq t}\Psi_{t,j}(\mathcal{A})$. Furthermore, we have $\lambda\in\bigcup_{i\in N}\bigcap_{j\in N, j\neq i}\Psi _{i,j}(\mathcal{A})$. □

Next, a comparison theorem is given for Theorem 1, Theorem 2 and Theorem 3.

### Theorem 4

*Let* $\mathcal{A}=(a_{i_{1}\cdots i_{m}})\in{\mathbb{R}}^{[m,n]}$. *Then*

$$ \Psi(\mathcal{A})\subseteq\mathcal{L}(\mathcal{A})\subseteq\mathcal {K}( \mathcal{A}). $$

### Proof

By Corollary 3.1 in [1], $\mathcal{L}(\mathcal{A})\subseteq \mathcal{K}(\mathcal{A})$ holds. Here, we only prove $\Psi(\mathcal{A})\subseteq\mathcal{L}(\mathcal{A})$. Let $z\in\Psi(\mathcal{A})$. Then there exists $i\in N$, such that $z\in\Psi_{i,j}(\mathcal{A})$, $\forall j\in N$, $j\neq i$, that is,

4 $$ \bigl(|z|-r_{i}^{\overline{\Delta}_{j}}(\mathcal{A})\bigr)|z|\leq r_{i}^{\Delta _{j}}(\mathcal{A})R_{j}(\mathcal{A}), \quad \forall j\in N, j\neq i. $$

Next, we divide our subject in two cases to prove $\Psi(\mathcal {A})\subseteq\mathcal{L}(\mathcal{A})$.

Case I: If $r_{i}^{\Delta_{j}}(\mathcal{A})R_{j}(\mathcal{A})=0$, then we have

$$ \bigl(|z|-\bigl(R_{i}(\mathcal{A})-|a_{ij\cdots j}|\bigr) \bigr)|z| \leq \bigl(|z|-r_{i}^{\overline{\Delta}_{j}}(\mathcal{A})\bigr)|z| \leq r_{i}^{\Delta_{j}}(\mathcal{A})R_{j}(\mathcal{A})=0 \leq|a_{ij\cdots j}|R_{j}(\mathcal{A}), $$

which implies that $z\in\bigcap_{j\in N, j\neq i}\mathcal{L}_{i,j}(\mathcal{A})\subseteq \mathcal{L}(\mathcal{A})$ from the arbitrariness of *j*, consequently, $\Psi(\mathcal{A})\subseteq\mathcal{L}(\mathcal{A})$.

Case II: If $r_{i}^{\Delta_{j}}(\mathcal{A})R_{j}(\mathcal{A})>0$, then dividing both sides by $r_{i}^{\Delta_{j}}(\mathcal{A})R_{j}(\mathcal {A})$ in (4), we have

5 $$ \frac{|z|-r_{i}^{\overline{\Delta}_{j}}(\mathcal{A})}{r_{i}^{\Delta _{j}}(\mathcal{A})} \frac{|z|}{R_{j}(\mathcal{A})}\leq1, $$

which implies

6 $$ \frac{|z|-r_{i}^{\overline{\Delta}_{j}}(\mathcal{A})}{r_{i}^{\Delta _{j}}(\mathcal{A})}\leq1, $$

or

7 $$ \frac{|z|}{R_{j}(\mathcal{A})}\leq1. $$

Let $a=|z|$, $b=r_{i}^{\overline{\Delta}_{j}}(\mathcal{A})$, $c=r_{i}^{\Delta _{j}}(\mathcal{A})-|a_{ij\cdots j}|$ and $d=|a_{ij\cdots j}|$. When (6) holds and $d=|a_{ij\cdots j}|>0$, from Lemma 2.2 in [11], we have

8 $$ \frac{|z|-(R_{i}(\mathcal{A})-|a_{ij\cdots j}|)}{|a_{ij\cdots j}|}=\frac {a-(b+c)}{d} \leq\frac{a-b}{c+d}= \frac{|z|-r_{i}^{\overline{\Delta}_{j}}(\mathcal {A})}{r_{i}^{\Delta_{j}}(\mathcal{A})}. $$

Furthermore, from (5) and (8), we have

$$ \frac{|z|-(R_{i}(\mathcal{A})-|a_{ij\cdots j}|)}{|a_{ij\cdots j}|}\frac {|z|}{R_{j}(\mathcal{A})} \leq\frac{|z|-r_{i}^{\overline{\Delta}_{j}}(\mathcal{A})}{r_{i}^{\Delta _{j}}(\mathcal{A})}\frac{|z|}{R_{j}(\mathcal{A})}\leq1, $$

equivalently,

$$ \bigl(|z|-\bigl(R_{i}(\mathcal{A})-|a_{ij\cdots j}|\bigr) \bigr)|z| \leq|a_{ij\cdots j}|R_{j}(\mathcal{A}), $$

which implies that $z\in\bigcap_{j\in N, j\neq i}\mathcal{L}_{i,j}(\mathcal{A})\subseteq \mathcal{L}(\mathcal{A})$ from the arbitrariness of *j*, consequently, $\Psi(\mathcal{A})\subseteq\mathcal{L}(\mathcal{A})$. When (6) holds and $d=|a_{ij\cdots j}|=0$, we have

$$ |z|-r_{i}^{\overline{\Delta}_{j}}(\mathcal{A})-r_{i}^{\Delta_{j}}( \mathcal {A})\leq0,\quad \textit{i.e.},\quad |z|-\bigl(R_{i}( \mathcal{A})-|a_{ij\cdots j}|\bigr)\leq0, $$

and furthermore

$$ \bigl(|z|-\bigl(R_{i}(\mathcal{A})-|a_{ij\cdots j}|\bigr) \bigr)|z| \leq0=|a_{ij\cdots j}|R_{j}(\mathcal{A}). $$

This also implies $\Psi(\mathcal{A})\subseteq\mathcal{L}(\mathcal{A})$.

On the other hand, when (7) holds, we only prove $\Psi (\mathcal{A})\subseteq\mathcal{L}(\mathcal{A})$ under the case that

9 $$ \frac{|z|-r_{i}^{\overline{\Delta}_{j}}(\mathcal{A})}{r_{i}^{\Delta _{j}}(\mathcal{A})}>1. $$

From (9), we have $\frac{a}{b+c+d}=\frac{|z|}{R_{i}(\mathcal{A})}>1$. When (7) holds and $|a_{ji\cdots i}|>0$, by Lemma 2.3 in [11], we have

10 $$ \frac{|z|}{R_{i}(\mathcal{A})}=\frac{a}{b+c+d} \leq\frac{a-b}{c+d}= \frac{|z|-r_{i}^{\overline{\Delta}_{j}}(\mathcal {A})}{r_{i}^{\Delta_{j}}(\mathcal{A})}. $$

By (7), Lemma 2.2 in [11] and similar to the proof of (8), we have

11 $$ \frac{|z|-(R_{j}(\mathcal{A})-|a_{ji\cdots i}|)}{|a_{ji\cdots i}|} \leq \frac{|z|}{R_{j}(\mathcal{A})}. $$

Multiplying (10) and (11), we have

$$ \frac{|z|-(R_{j}(\mathcal{A})-|a_{ji\cdots i}|)}{|a_{ji\cdots i}|}\frac {|z|}{R_{i}(\mathcal{A})} \leq \frac{|z|-r_{i}^{\overline{\Delta}_{j}}(\mathcal{A})}{r_{i}^{\Delta _{j}}(\mathcal{A})}\frac{|z|}{R_{j}(\mathcal{A})} \leq1; $$

equivalently,

$$ \bigl(|z|-\bigl(R_{j}(\mathcal{A})-|a_{ji\cdots i}|\bigr) \bigr)|z| \leq|a_{ji\cdots i}|R_{i}(\mathcal{A}). $$

This implies $z\in\bigcap_{i\in N, i\neq j}\mathcal{L}_{j,i}(\mathcal {A})\subseteq\mathcal{L}(\mathcal{A})$ and $\Psi(\mathcal{A})\subseteq \mathcal{L}(\mathcal{A})$ from the arbitrariness of *i*. When (7) holds and $|a_{ji\cdots i}|=0$, we can obtain

$$ |z|-R_{j}(\mathcal{A})\leq0,\quad \textit{i.e.},\quad |z|- \bigl(R_{j}(\mathcal{A})-|a_{ji\cdots i}|\bigr)\leq0 $$

and

$$ \bigl(|z|-\bigl(R_{j}(\mathcal{A})-|a_{ji\cdots i}|\bigr) \bigr)|z| \leq0=|a_{ji\cdots i}|R_{i}(\mathcal{A}). $$

This also implies $\Psi(\mathcal{A})\subseteq\mathcal{L}(\mathcal{A})$. The conclusion follows from Case I and Case II. □

### Remark 1

Theorem 4 shows that the set $\Psi(\mathcal{A})$ in Theorem 3 is tighter than $\mathcal{K}(\mathcal{A})$ in Theorem 1 and $\mathcal{L}(\mathcal{A})$ in Theorem 2, that is, $\Psi(\mathcal{A})$ can capture all *Z*-eigenvalues of $\mathcal{A}$ more precisely than $\mathcal{K}(\mathcal{A})$ and $\mathcal{L}(\mathcal{A})$.

Now, we give an example to show that $\Psi(\mathcal{A})$ is tighter than $\mathcal{K}(\mathcal{A})$ and $\mathcal{L}(\mathcal{A})$.

### Example 1

Let $\mathcal{A}=(a_{ijkl})\in{\mathbb{R}}^{[4,2]}$ be a symmetric tensor defined by

$$a_{1222}=1,\qquad a_{2222}=2, \quad \mbox{and}\quad a_{ijkl}=0\quad \mbox{elsewhere}. $$

By computation, we see that all the *Z*-eigenvalues of $\mathcal{A}$ are −0.5000, 0 and 2.7000. By Theorem 1, we have

$$\begin{aligned} \mathcal{K}(\mathcal{A}) =&\mathcal{K}_{1}(\mathcal{A})\cup\mathcal {K}_{2}(\mathcal{A}) =\bigl\{ z\in{\mathbb{C}}: \vert z \vert \leq1 \bigr\} \cup\bigl\{ z\in{\mathbb{C}}: \vert z \vert \leq5\bigr\} \\ =&\bigl\{ z\in{ \mathbb{C}}: \vert z \vert \leq5\bigr\} . \end{aligned}$$

By Theorem 2, we have

$$\begin{aligned} \mathcal{L}(\mathcal{A}) =&\mathcal{L}_{1,2}(\mathcal{A})\cup\mathcal {L}_{2,1}(\mathcal{A}) =\bigl\{ z\in{\mathbb{C}}: \vert z \vert \leq2.2361\bigr\} \cup\bigl\{ z\in{\mathbb{C}}: \vert z \vert \leq 5\bigr\} \\ =& \bigl\{ z\in{\mathbb{C}}: \vert z \vert \leq5\bigr\} . \end{aligned}$$

By Theorem 3, we have

$$\begin{aligned} \Psi(\mathcal{A}) =&\Psi_{1,2}(\mathcal{A})\cup\Psi_{2,1}( \mathcal{A}) =\bigl\{ z\in{\mathbb{C}}: \vert z \vert \leq2.2361\bigr\} \cup \bigl\{ z\in{\mathbb{C}}: \vert z \vert \leq 3\bigr\} \\ =&\bigl\{ z\in{\mathbb{C}}: \vert z \vert \leq3\bigr\} . \end{aligned}$$

**Figure 1:**
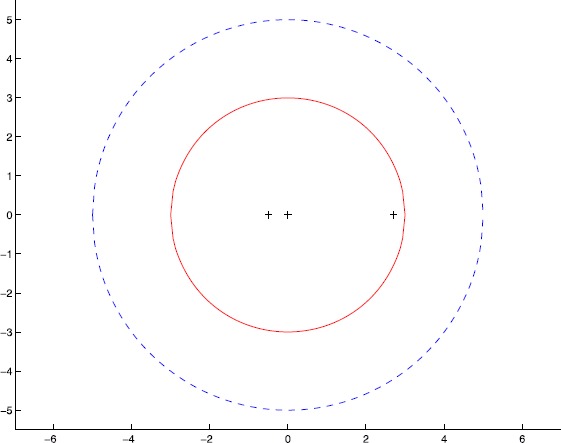
**Comparisons of** $\pmb{\mathcal{K}(\mathcal{A})}$ **,** $\pmb{\mathcal {L}(\mathcal{A})}$ **and** $\pmb{\Psi(\mathcal{A})}$ **.**

The *Z*-eigenvalue inclusion sets $\mathcal{K}(\mathcal{A})$, $\mathcal {L}(\mathcal{A})$, $\Psi(\mathcal{A})$ and the exact *Z*-eigenvalues are drawn in Figure 1, where $\mathcal{K}(\mathcal{A})$ and $\mathcal{L}(\mathcal{A})$ are represented by blue dashed boundary, $\Psi(\mathcal{A})$ is represented by red solid boundary and the exact eigenvalues are plotted by ‘+’, respectively. It is easy to see $\sigma(\mathcal{A})\subseteq\Psi(\mathcal {A})\subset\mathcal{L}(\mathcal{A})\subseteq\mathcal{K}(\mathcal{A})$, that is, $\Psi(\mathcal{A})$ can capture all *Z*-eigenvalues of $\mathcal{A}$ more precisely than $\mathcal{L}(\mathcal{A})$ and $\mathcal{K}(\mathcal{A})$.

## A new upper bound for the *Z*-spectral radius of weakly symmetric nonnegative tensors

As an application of the results in Section 2, we in this section give a new upper bound for the *Z*-spectral radius of weakly symmetric nonnegative tensors.

### Theorem 5

*Let* $\mathcal{A}=(a_{i_{1}\cdots i_{m}})\in{\mathbb{R}}^{[m,n]}$ *be a weakly symmetric nonnegative tensor*. *Then*

$$ \varrho(\mathcal{A})\leq\max_{i\in N}\min_{j\in N, j\neq i} \Phi _{i,j}(\mathcal{A}), $$

*where*

$$ \Phi_{i,j}(\mathcal{A})=\frac{1}{2} \Bigl\{ r_{i}^{\overline{\Delta}_{j}}( \mathcal{A})+\sqrt{\bigl(r_{i}^{\overline{\Delta }_{j}}(\mathcal{A}) \bigr)^{2}+4r_{i}^{\Delta_{j}}(\mathcal{A})R_{j}( \mathcal{A})} \Bigr\} . $$

### Proof

From Lemma 4.4 in [1], we know that $\varrho(\mathcal{A})$ is the largest *Z*-eigenvalue of $\mathcal{A}$. It follows from Theorem 3 that there exists $i\in N$ such that

12 $$ \bigl(\varrho(\mathcal{A})-r_{i}^{\overline{\Delta}_{j}}( \mathcal{A}) \bigr)\varrho(\mathcal{A})\leq r_{i}^{\Delta_{j}}( \mathcal{A})R_{j}(\mathcal{A}),\quad \forall j\in N, j\neq i. $$

Solving $\varrho(\mathcal{A})$ in (12) gives

$$ \varrho(\mathcal{A})\leq\frac{1}{2} \Bigl\{ r_{i}^{\overline{\Delta}_{j}}( \mathcal{A})+\sqrt{\bigl(r_{i}^{\overline{\Delta }_{j}}(\mathcal{A}) \bigr)^{2}+4r_{i}^{\Delta_{j}}(\mathcal{A})R_{j}( \mathcal{A})} \Bigr\} =\Phi_{i,j}(\mathcal{A}). $$

From the arbitrariness of *j*, we have $\varrho(\mathcal{A})\leq\min_{j\in N, j\neq i}\Phi_{i,j}(\mathcal{A})$. Furthermore, $\varrho(\mathcal{A})\leq\max_{i\in N}\min_{j\in N, j\neq i}\Phi _{i,j}(\mathcal{A})$. □

By Theorem 4, Theorem 4.5 and Corollary 4.1 in [1], the following comparison theorem can be derived easily.

### Theorem 6

*Let* $\mathcal{A}=(a_{i_{1}\cdots i_{m}})\in{\mathbb{R}}^{[m,n]}$ *be a weakly symmetric nonnegative tensor*. *Then the upper bound in Theorem* 5 *is sharper than those in Theorem *4.5 *of* [1] *and Corollary *4.5 *of* [5], *that is*,

$$\begin{aligned} \varrho(\mathcal{A}) \leq&\max_{i\in N}\min_{j\in N, j\neq i} \Phi _{i,j}(\mathcal{A}) \\ \leq&\max_{i\in N}\min_{j\in N, j\neq i}\frac{1}{2} \bigl\{ R_{i}(\mathcal{A})-a_{ij\cdots j}+\sqrt{ \bigl(R_{i}(\mathcal{A})-a_{ij\cdots j}\bigr)^{2}+4a_{ij\cdots j}R_{j}( \mathcal{A})} \bigr\} \\ \leq&\max_{i\in N}R_{i}(\mathcal{A}). \end{aligned}$$

Finally, we show that the upper bound in Theorem 5 is sharper than those in [1, 5–8, 10] by the following example.

### Example 2

Let $\mathcal{A}=(a_{ijk})\in{\mathbb{R}}^{[3,3]}$ with the entries defined as follows:

$$\begin{aligned}& \mathcal{A}(:,:,1)=\left ( \textstyle\begin{array}{@{}c@{\quad}c@{\quad}c@{}} 3&3&0\\ 3&2&2.5\\ 0.5&2.5&0 \end{array}\displaystyle \right ),\qquad \mathcal{A}(:,:,2)=\left ( \textstyle\begin{array}{@{}c@{\quad}c@{\quad}c@{}} 3&2&2\\ 2&0&3\\ 2.5&3&1 \end{array}\displaystyle \right ), \\& \mathcal{A}(:,:,3)=\left ( \textstyle\begin{array}{@{}c@{\quad}c@{\quad}c@{}} 1&3&0\\ 2.5&3&1\\ 0&1&0 \end{array}\displaystyle \right ). \end{aligned}$$

It is not difficult to verify that $\mathcal{A}$ is a weakly symmetric nonnegative tensor. By both Corollary 4.5 of [5] and Theorem 3.3 of [6], we have

$$\varrho(\mathcal{A})\leq19. $$

By Theorem 3.5 of [7], we have

$$\varrho(\mathcal{A})\leq18.6788. $$

By Theorem 4.6 of [1], we have

$$\varrho(\mathcal{A})\leq18.6603. $$

By both Theorem 4.5 of [1] and Theorem 6 of [8], we have

$$\varrho(\mathcal{A})\leq18.5656. $$

By Theorem 4.7 of [1], we have

$$\varrho(\mathcal{A})\leq18.3417. $$

By Theorem 2.9 of [10], we have

$$\varrho(\mathcal{A})\leq17.2063. $$

By Theorem 5, we obtain

$$\varrho(\mathcal{A})\leq15.2580, $$

which shows that the upper bound in Theorem 5 is sharper.

## Conclusions

In this paper, we present a new *Z*-eigenvalue localization set $\Psi (\mathcal{A})$ and prove that this set is tighter than those in [1]. As an application, we obtain a new upper bound $\max_{i\in N}\min_{j\in N, j\neq i}\Phi_{i,j}(\mathcal{A})$ for the *Z*-spectral radius of weakly symmetric nonnegative tensors, and we show that this bound is sharper than those in [1, 5–8, 10] in some cases by a numerical example.
